# Targeting Lysosomes in Cancer as Promising Strategy to Overcome Chemoresistance—A Mini Review

**DOI:** 10.3389/fonc.2020.01156

**Published:** 2020-07-09

**Authors:** Franz Geisslinger, Martin Müller, Angelika M. Vollmar, Karin Bartel

**Affiliations:** Pharmaceutical Biology, Department Pharmacy, Ludwig-Maximilians-University of Munich, Munich, Germany

**Keywords:** chemoresistance, cytostatics, cancer, lysosome, TFEB

## Abstract

To date, cancer remains a worldwide leading cause of death, with a still rising incidence. This is essentially caused by the fact, that despite the abundance of therapeutic targets and treatment strategies, insufficient response and multidrug resistance frequently occur. Underlying mechanisms are multifaceted and extensively studied. In recent research, it became evident, that the lysosome is of importance in drug resistance phenotypes. While it has long been considered just as cellular waste bag, it is now widely known that lysosomes play an important role in important cellular signaling processes and are in the focus of cancer research. In that regard lysosomes are now considered as so-called “drug safe-houses” in which chemotherapeutics are trapped passively by diffusion or actively by lysosomal P-glycoprotein activity, which prevents them from reaching their intracellular targets. Furthermore, alterations in lysosome to nucleus signaling by the transcription factor EB (TFEB)—mTORC1 axis are implicated in development of chemoresistance. The identification of lysosomes as essential players in drug resistance has introduced novel strategies to overcome chemoresistance and led to innovate therapeutic approaches. This mini review gives an overview of the current state of research on the role of lysosomes in chemoresistance, summarizing underlying mechanisms and treatment strategies and critically discussing open questions and drawbacks.

## Introduction

Cancer is still the second leading cause of death worldwide. In total, the annual number of new cancer diagnoses is 18 million with rising incidence and about 25–30% of all deaths worldwide are connected to cancer and cancer-related diseases (1, 2). Cancer cells possess several features distinct from healthy cells, described as the hallmarks of cancer, which enable them to survive and constantly proliferate (3).

Lysosomes are part of the endolysosomal system (ES), which has long simply been considered as the cell's recycling or waste compartment. Interestingly, the ES was discovered to be also important for sustaining several cancer hallmarks, including migration of invasive cancer cells and neoangiogenesis of endothelial cells (4–7). The ES consists of early, late and recycling endosomes as well as lysosomes, which are separated from the cytosol by a lipid bilayer (69). The ES biogenesis is predominantly regulated by TFEB and mTORC1 signaling and frequently altered upon oncogenic transformation. ES organelles are distinguished by their characteristic intraluminal composition, acidic pH and expression of surface proteins (8, 9). In particular, the lysosome contains various hydrolases, such as proteases and lipases, and displays a luminal pH of about 4.5–5. Today it is recognized as important regulator of nutrient homeostasis, apoptosis, autophagy and membrane trafficking, processes cancer cells critically depend on (7, 69). Consequently, targeting lysosomes gained interest in cancer therapy. Current research is focused on several of its membrane proteins, e.g., the V-ATPase and lysosomal cation channels, like TRPMLs and TPCs, for which excellent reviews are available (9–13).

A major drawback in cancer therapy is the phenomenon of multidrug resistance (MDR), in which tumor cells become unresponsive to treatment, despite the availability of a high variety of targets and related treatment strategies (14–16). The underlying mechanisms are very versatile. A prominent pathway, however, is linked to aberrant drug efflux mediated by the drug transporter P-glycoprotein (P-gp), an ATP-dependent efflux pump (17). Despite intensive research, all clinical trials evaluating P-gp inhibitors failed to date (18).

This review is divided into two parts. Firstly, we critically discuss literature connecting lysosomes and chemoresistance and secondly, we provide an overview of lysosome-based treatment strategies to overcome drug resistance.

## Lysosomes and Chemoresistance

Notably, the lysosome recently emerged as promising target to overcome chemoresistance, as increasing evidence suggests that it is involved in P-gp trafficking, serves as drug safe house and links lysosomal biogenesis, induced by transcription factor TFEB, to chemoresistance phenotypes (19–21). Therefore, targeting lysosomal function might improve response to chemotherapy as explained in detail below.

### Lysosomal Function Is Crucial for P-Glycoprotein Trafficking

P-gp is a membrane-bound protein, usually embedded in the plasma membrane, actively transporting cargo into the extracellular space. Recently, in resistant cancer cells, lysosomal overexpression of P-gp has been reported. Mechanistically, it is hypothesized that P-gp is incorporated into lysosomal membranes during trafficking and recycling events, rather than being redistributed after *de-novo* synthesis (19, 20, 22).

The ES is substantially involved in protein trafficking, a process that is dependent on proper function of ES membrane-integrated proteins and ion channels (5, 23, 24). Disturbance of ES membrane protein function in cancer cells is a promising treatment strategy. For instance, V-ATPase inhibition induces lysosomal alkalization and subsequently disrupts receptor trafficking (24–26). Additionally, inhibiting TPC2 and TRPML1 ion channels also impairs trafficking and induces apoptosis (5, 27). Extensively studied players of regulation of vesicular trafficking are the RabGTPases. This large family of small GTPases controls membrane identity, vesicle budding, uncoating, motility and fusion during vesicular trafficking (28–32). Rab GTPases show a distinct intracellular distribution pattern of different members to the respective organelle, thereby regulating directed vesicle trafficking. Lysosome associated Rab GTPases are e.g., Rab4, Rab5, and Rab7 (33). Although their impacts on P-gp trafficking are controversial and dependent on the cell type, Rab4 and Rab5 were shown to affect intracellular localization of P-gp (34). Nevertheless, Ferrandiz-Huertas et al. (35) were able to demonstrate that overexpression of Rab4 leads to a decrease in membranous P-gp abundance and subsequently to increased intracellular daunorubicin concentrations. Furthermore, Rab4 levels seem to correlate with resistance status in this study, as resistant cells have decreased Rab4 levels as compared to the parental cell line (35).

**Figure 1:**
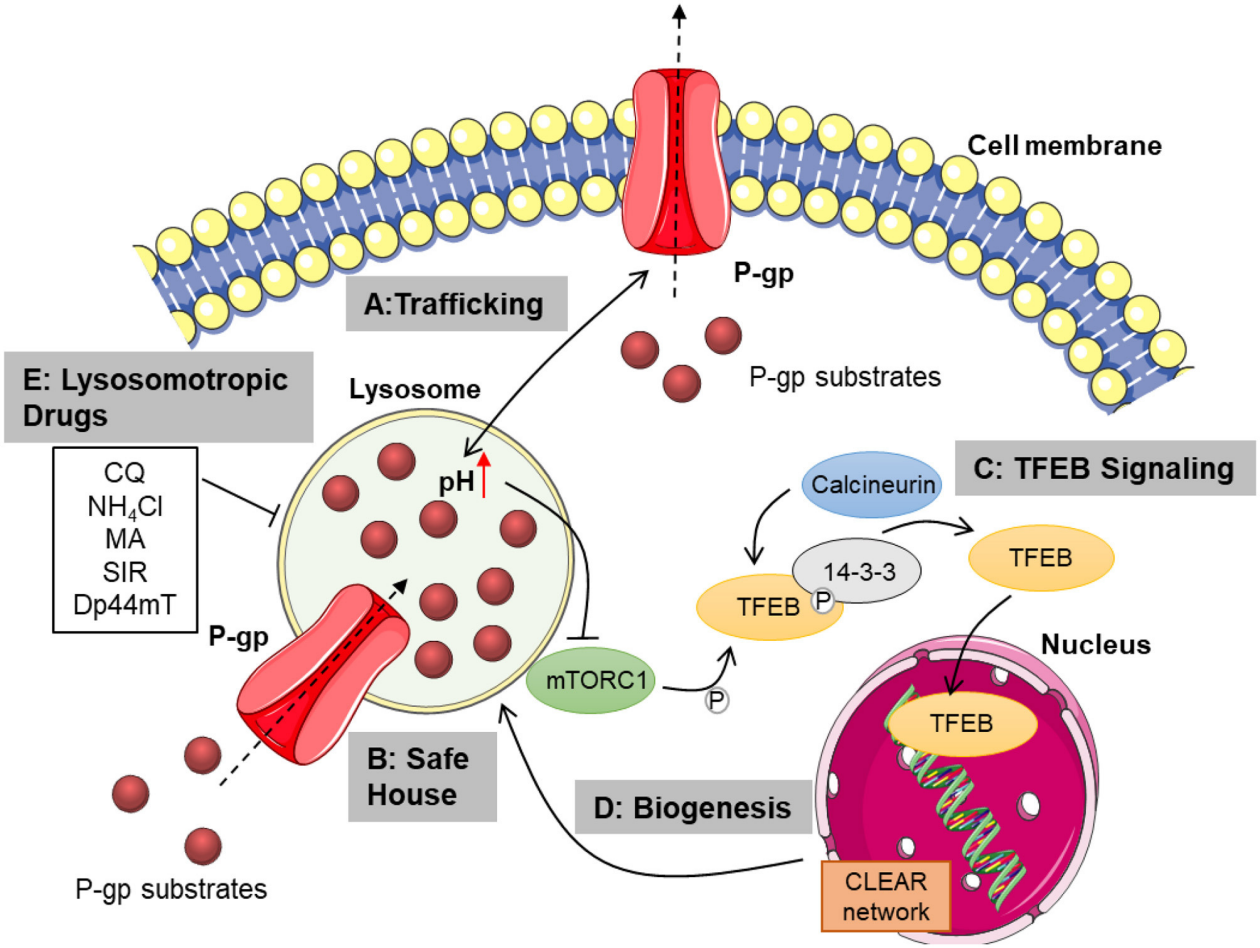
Overview of lysosomal mechanisms contributing to chemoresistance. Lysosomal function is pivotal for proper trafficking of P-glycoprotein to the cell membrane. Membranous P-gp transports cytostatics from the cytosol to the extracellular space **(A)**. Lysosomal P-gp pumps its substrates into the lysosomal lumen, where they are sequestered in dependence of their physicochemical properties. Passive diffusion of hydrophobic weak bases also contributes to lysosomal drug sequestration **(B)**. Subsequent disturbance of lysosomal function leads to TFEB activation mediated by mTORC1 inhibition and calcineurin activation. TFEB is then released from 14-3-3 and translocates to the nucleus, transcribing genes from the CLEAR network **(C)**. This promotes lysosomal biogenesis, increasing lysosomal mass and thus sequestration capacity **(D)**. Inhibiting lysosomal function by treatment with lysosomotropic or lysosome damaging agents as well as elevating lysosomal pH, may overcome chemoresistance mediated by lysosomal drug sequestration **(E)**. CQ, chloroquine; NH_4_Cl, ammonium chloride; MA, methylamine; SIR, siramesine; Dp44mT, Di-2-pyridylketone-4,4-dimethyl-3-thiosemicarbazone; P-gp, P-glycoprotein; mTORC1, mammalian target of rapamycin complex 1; TFEB, transcription factor EB; CLEAR, coordinated lysosomal expression and regulation; P, phosphate. This figure was created using images from Servier Medical Art Commons Attribution 3.0 Unported License. (http://smart.servier.com). Servier Medical Art by Servier is licensed under a Creative Commons Attribution 3.0 Unported License.

Based on these studies, evidence is given that modulating endolysosomal function impairs P-gp membrane trafficking and recycling. Hence, targeting the ES might be a reasonable approach to overcome multidrug resistance (Pathway A in Figure 1). However, most of the currently available studies remain to prove a therapeutic benefit and the effects are strongly cell type dependent.

### The Lysosome as Drug Safe House

An important lysosomal mechanism contributing to chemoresistance is the so-called drug safe house effect. The acidity of lysosomes facilitates luminal accumulation of cytostatic weak bases and leads to their protonation, reducing their ability to pass the lysosomal membrane, causing lysosomal drug sequestration (36). Upon lysosomal trapping, cytostatics are prevented from reaching their intracellular targets, which are usually located in the nucleus or the cytosol and therefore fail to exert cytotoxicity (37) (Pathway B in Figure 1). Cytostatics enter the lysosome either by passive diffusion along the pH gradient or may be actively transported across the membrane by inward turned P-gp drug efflux pumps embedded in the lysosomal membrane. Lysosomal sequestration capacity therefore greatly depends on the physicochemical properties of the cytostatic and on the lysosomal features like pH and lysosomal volume (19, 37). Typically, weak bases can enter the lysosome via passive diffusion and are trapped upon protonation, yet also hydrophilicity of the protonated or deprotonated drug is an important factor as it strongly correlates with membrane permeability (19). In that regard, pKa values, which display acidity or basicity of a compound and logP/D values, which represent hydrophilicity of non-ionizable and ionizable molecules, respectively, can be used to estimate lysosomal sequestration of a drug (38, 39). For an active lysosomal trapping, the abundance of lysosomal P-gp activity is a determining factor. As discussed above, P-gp can be trafficked to the lysosomal membrane under stress conditions, which has been reported to tremendously enhance resistance to cytostatic P-gp substrates (20). In turn, stress factors like reactive oxygen species can be induced by cytostatics, further increasing lysosomal P-gp abundance and hence resistance.

However, the interplay between lysosomal properties, active or passive drug accumulation and effective sequestration are complex and cell line dependent. For instance, the weak base doxorubicin undergoes lysosomal trapping in UMUC-3 cells, but not in KB31 cells, most likely as result of different lysosomal properties. Interestingly, in the P-gp overexpressing KB31-subline KBV1, doxorubicin is effectively sequestered, an effect which is reversed by P-gp inhibition (19). Moreover, lysosomes of resistant cell lines often show a more acidic pH than their parental cell lines, probably further enhancing drug sequestration capability (40). Yet, the non-basic and lipophilic P-gp substrate paclitaxel is neither sequestered in KB31 nor KBV1 cells, owing to its ability to freely permeate the lysosomal membrane (19).

The fate of drugs sequestered in lysosomes remains to be clearly elucidated. It is suggested that they either stay trapped in lysosomes or are eliminated from the cell by drug-induced lysosomal exocytosis, preventing lysosomal damage. Supporting the hypothesis of lysosomal exocytosis, treatment with cytostatics leads to redistribution of basally perinuclear lysosomes to the plasma membrane. Furthermore, lysosomal content is increasingly released into the extracellular compartment, such as cathepsin D and V-ATPase (41, 71). Enhanced membrane fusion and subsequent exocytosis could be mediated by lysosomal calcium release via activation of lysosomal cation channels, such as TRPML1 (42). After lysosomal exocytosis, former trapped cytostatics are again abundant in the extracellular compartment, enabling repeated diffusion into the cell. Therefore, it is doubtful whether this mechanism contributes to chemoresistance, requiring further investigation. Nevertheless, the implication of lysosomes in chemoresistance is not restricted to its role as “drug safe house.” In fact, lysosomal abundance of cytostatics leads to changes in lysosomal properties, including pH and lysosomal volume, further influencing lysosomal signaling.

### TFEB Signaling Regulating Drug Resistance

Lysosomal properties are mainly regulated by the transcription factor EB (TFEB), the master regulator of lysosomal biogenesis, which when activated transcribes genes belonging to the CLEAR (coordinated lysosomal expression and regulation) network. TFEB is usually retained inactive in the cytosol, by binding to the regulatory protein 14-3-3, which prevents nuclear translocation. The binding of TFEB to 14-3-3 depends on its phosphorylation status, which is regulated by the kinase mTORC1 and the phosphatase calcineurin. mTORC1 phosphorylates TFEB facilitating binding, while calcineurin dephosphorylates TFEB, leading to dissociation and subsequent nuclear translocation (43, 44).

By accumulating in the lysosomal lumen, cytostatic weak bases act like classic lysosomotropic compounds, raising lysosomal pH and increasing lysosomal volume. Lysosomotropism is the propensity of a typically basic substance to specifically accumulate in lysosomes and consequently alter lysosomal features and induce lysosomal membrane permeabilization (45). In particular, an increase of lysosomal volume after treatment with the cytostatic weak base sunitinib, but not with the non-basic cytostatics 5-fluorouracil and pemetrexed, positively correlates with drug resistance. In detail, treatment with cytostatic weak bases, such as trametinib, vincristine, sunitinib and doxorubicin leads to increased lysosomal volume in several cancer cell lines (39, 46, 71). Furthermore, a different study shows that LAMP1, a lysosomal marker, is upregulated in breast cancer cells upon development of resistance by continuous exposure to doxorubicin (47).

In line with an increase in lysosomal volume, TFEB is activated when exposed to cytostatic weak bases and upregulated upon development of chemoresistance, as shown for doxorubicin or mitoxantrone treatment. Further, mTORC1 activity is inhibited upon treatment with the cytostatic weak bases sunitinib and siramesine, while calcineurin activity is enhanced, leading to TFEB activation (21, 39) (Pathway C in Figure 1). Causal for mTORC1 inhibition might be a change in lysosomal properties, especially pH. If lysosomal pH is increased, for instance by inhibition of the V-ATPase, a proton pump maintaining lysosomal acidity, TFEB is activated and translocated to the nucleus (43). Weak base cytostatics accumulating in lysosomes rise lysosomal pH in a similar manner, as shown for doxorubicin in cardiomyocytes, for example (48). Consequently, TFEB and lysosomes could build a bidirectional axis with lysosomal drug accumulation activating TFEB and TFEB inducing lysosomal biogenesis, which increases lysosomal sequestration capacity and exerts a feedback loop (Pathway D in Figure 1). Additionally, this feedback loop might be enhanced by the influence of lysosomal calcium signaling. TFEB regulation is closely linked to lysosomal calcium signaling through the TRPML1 ion channel. Upon lysosomal stress, TRPML1 channels release calcium into the cytosol, creating a calcium enriched microenvironment in which the calcium-dependent phosphatase calcineurin is activated and dephosphorylates TFEB (44, 49). Increasing evidence suggests that TRPML1 channel function is important for cancer cells as genetic depletion leads to decreased cell survival and it has also been reported that TRPML1 and mTORC1 signaling are essential for aggressive cancer cells (50–52). Thus, TFEB and its regulators mTORC1 and calcineurin are considered as potential target for drug resistant tumors. However, which mechanism of those explained above contributes to the sensitization effect remains unknown and further studies addressing this topic are currently missing.

Furthermore, TFEB acts as transcription factor for several proteins essential for autophagy, a complex process promoting cell survival during stress conditions (53). Autophagy is a physiological “self-eating” recycling process which removes defective proteins or organelles, to maintain cellular homeostasis. In cancer cells, autophagy is a paradox which is on the one hand able to prevent tumor initiation by preventing cell damage, but on the other driving tumor progression by facilitating adaptations to stress conditions like nutrient deprivation and hypoxia (54). There is furthermore growing evidence that autophagy is a driving factor for chemoresistance. Autophagic signaling is also altered upon treatment with cytostatics, namely vincristine and doxorubicin, indicated by enhanced LC3-II and p62 levels, protein markers used for visualizing inhibited autophagy. Subsequent promotion of cell survival can be inhibited by knockdown of Atg5, an important regulator of autophagosome formation (46, 47, 55). Evidence further suggests a protective role for autophagy regulators ATG3, 5, 6, 7, and 12 in resistant cells, however, detailed mechanisms are not yet fully understood and hence require further studies (56–60). Interestingly, besides TFEB-regulated lysosomal alterations upon treatment with cytostatics, TFEB also contributes to cell survival by mechanisms independent of the aforementioned. For instance, TFEB influences DNA repair, leading to inhibition of apoptosis. Therefore, knockdown of TFEB prevents DNA repair and thus sensitizes MDA-MB-231 cells to doxorubicin treatment, promoting cell death induction (70). Brady et al. hypothesize that this TFEB-dependent DNA repair mechanism is mediated by p53-related signaling and is, besides TFEB, also activated by TFE3, a transcription factor closely related to TFEB (61). Therefore, lysosomal proteins not only favor resistance by drug sequestration being the “final destination” for cytostatics, but they also serve as signaling hub, activating cell survival pathways.

## Lysosome-Based Treatment Strategies

Contribution of lysosomes to chemoresistance raised interest in lysosome-targeting strategies to sensitize tumor cells to chemotherapy. These strategies mainly focus on lysosomotropic compounds, which act by accumulating in the lysosomal lumen, thereby elevating lysosomal pH because of their weak base characteristics and usually inducing lysosomal membrane permeabilization (45). Lysosomotropic compounds commonly used in *in vitro* experiments are chloroquine, ammonium chloride, methylamine and siramesine (19, 62). By inhibiting lysosomal drug sequestration as consequence of pH elevation, lysosomotropic adjuvants cause intracellular redistribution of cytostatics from the lysosomal lumen to the cytosol and subsequently to their sites of action (Pathway E in Figure 1). This opens the possibility for combination therapies to overcome resistance. Indeed, combination of chloroquine, ammonium chloride or methylamine strikingly sensitizes resistant KBV1 cells to treatment with vincristine and doxorubicin. Additionally, combining vincristine with siramesine is superior to monotherapy *in vitro* and *in vivo* in breast cancer (19, 46).

As these data suggest a synergistic effect of cytostatics and inhibition of lysosomal function, also the V-ATPase might be considered as reasonable target due to its implication in sustaining lysosomal acidity. Evidence suggests that V-ATPase overexpression is associated with chemoresistance and V-ATPase knockdown sensitizes doxorubicin-resistant MCF-7 cells to doxorubicin and vincristine (63). Yet, studies addressing pharmacological V-ATPase inhibition regarding chemoresistance are scarce, requiring further research.

Another interesting approach focuses on the experimental metal chelator Di-2-pyridylketone-4,4-dimethyl-3-thiosemicarbazone (Dp44mT). By accumulating within lysosomes and forming redox-active copper complexes, Dp44mT potently induces lysosomal damage and subsequently lysosomal cell death (64), which is characterized by cathepsin D release from the lysosome into the cytosol and subsequent mitochondrial release of cytochrome c, initiating apoptosis (65). Therefore, Dp44mT toxicity is dependent on its lysosomal trapping. As Dp44mT is a weak base P-gp substrate, it accumulates stronger in P-gp expressing, resistant cells, enabling specific killing of resistant cells, while sensitive cells are less affected (66). Furthermore, Dp44mT could be used in combination with basic cytostatics due to its inhibitory effect on lysosomal drug sequestration. In this context, targeting acid sphingomyelinase, a lipase responsible for lysosomal hydrolysis of sphingomyelin, was proven to be another effective strategy to successfully treat multidrug resistant tumors. Siramesine and numerous approved basic amphiphilic drugs, including tricyclic antidepressants and antihistamines, can disrupt lysosomal membrane integrity by inhibiting acid sphingomyelinase and thereby achieve cancer-specific cytotoxicity (62). Thus, introducing lysosomal membrane permeabilization exemplarily emphasizes the potential of lysosome-targeted approaches to overcome chemoresistance.

## Conclusion

Despite high efforts to improve chemotherapy, treatment failure and resistance mechanisms remain a major challenge. Exemplarily, for the efflux pump P-gp, which can limit efficacy of cytostatic drugs, no clinically approved treatment options are available to date. As summarized in this review, the contribution of lysosomes to drug resistance opened a new research field in the context of multidrug resistance to overcome chemoresistance. This implication is not restricted to direct lysosomal mechanisms, but also includes lysosome-associated signaling pathways. Evidence suggests that interfering with lysosomal function might be a promising approach enabling sensitization to chemotherapy, influencing several survival-promoting mechanisms, such as trafficking of efflux transporters, drug sequestration and pathways regulated by TFEB, including autophagy and DNA repair. Additionally, inhibition of lysosomal function could target P-glycoprotein-driven chemoresistance, giving hope for lysosome-targeted adjuvants in the future.

However, also lysosome-based strategies have their drawbacks. Targeting lysosomal function in general is not a cancer-specific approach, suggesting that it could lead to severe side effects, as lysosomal function is pivotal for virtually all types of cells, especially for immune cells (67, 68). Cancer-specific or cancer-enriched targets in this concern are still rare, but there is a high variety of lysosomal proteins, e.g., Rab GTPases, ion channels, lipases or the V-ATPase, which could serve as specific targets to disturb lysosomal function and thus drug resistance phenotypes of cancer cells more selectively. Therefore, further studies addressing the drawbacks and therapeutic potential of lysosome-based combination strategies are needed.

## Author Contributions

FG and KB conception. FG wrote the first draft of the manuscript. KB and MM wrote sections of the manuscript. AV and KB critically revised the manuscript. All authors contributed to the article and approved the submitted version.

## Conflict of Interest

The authors declare that the research was conducted in the absence of any commercial or financial relationships that could be construed as a potential conflict of interest.
